# SakA Regulates Morphological Development, Ochratoxin A Biosynthesis and Pathogenicity of *Aspergillus westerdijkiae* and the Response to Different Environmental Stresses

**DOI:** 10.3390/toxins15040292

**Published:** 2023-04-17

**Authors:** Peidong Si, Gang Wang, Wenqing Wu, Sarfaraz Hussain, Ling Guo, Wei Wu, Qingli Yang, Fuguo Xing

**Affiliations:** 1 College of Food Science and Engineering, Qingdao Agricultural University, Qingdao 266109, China; sipeidong@163.com (P.S.); wuweiouc@126.com (W.W.); rice407@163.com (Q.Y.); 2 Key Laboratory of Agro-Products Quality and Safety Control in Storage and Transport Process, Ministry of Agriculture and Rural Affairs, Institute of Food Science and Technology, Chinese Academy of Agricultural Sciences, Beijing 100193, China; wanggang02@caas.cn (G.W.); w18622586026@163.com (W.W.); sarfaraz.h@caas.cn (S.H.); guo13602004336@outlook.com (L.G.)

**Keywords:** *Aspergillus westerdijkiae*, mycotoxin, SakA, MAPK, environmental stress, ochratoxin A, virulence

## Abstract

Ochratoxin A (OTA), as a common mycotoxin, has seriously harmful effects on agricultural products, livestock and humans. There are reports on the regulation of SakA in the MAPK pathway, which regulates the production of mycotoxins. However, the role of SakA in the regulation of *Aspergillus westerdijkiae* and OTA production is not clear. In this study, a *SakA* deletion mutant (*ΔAwSakA*) was constructed. The effects of different concentrations of D-sorbitol, NaCl, Congo red and H_2_O_2_ on the mycelia growth, conidia production and biosynthesis of OTA were investigated in *A. westerdijkiae* WT and *ΔAwSakA*. The results showed that 100 g/L NaCl and 3.6 M D-sorbitol significantly inhibited mycelium growth and that a concentration of 0.1% Congo red was sufficient to inhibit the mycelium growth. A reduction in mycelium development was observed in *ΔAwSakA*, especially in high concentrations of osmotic stress. A lack of *AwSakA* dramatically reduced OTA production by downregulating the expression of the biosynthetic genes *otaA*, *otaY*, *otaB* and *otaD*. However, *otaC* and the transcription factor *otaR*1 were slightly upregulated by 80 g/L NaCl and 2.4 M D-sorbitol, whereas they were downregulated by 0.1% Congo red and 2 mM H_2_O_2_. Furthermore, *ΔAwSakA* showed degenerative infection ability toward pears and grapes. These results suggest that *AwSakA* is involved in the regulation of fungal growth, OTA biosynthesis and the pathogenicity of *A. westerdijkiae* and could be influenced by specific environmental stresses.

## 1. Introduction

Ochratoxin A (OTA) is a natural contaminant of grain, feed, fruits and their products. Since OTA was discovered in 1965 [1], multiple toxicities have been proved by researchers, such as nephrotoxic, hepatotoxic, embryotoxic, teratogenic, neurotoxic, genotoxic and carcinogenic toxicities [2]. In addition, it is important to note that OTA can produce cumulative toxicity in the body when ingested by humans or animals [3]. The International Agency for Research on Cancer (IARC) explicitly defined OTA as a group 2B carcinogen in 1993 [4]. As one of the most important mycotoxins, knowing the biosynthetic pathway and the regulation mechanisms of OTA production seems to be of great importance. 

OTA is a secondary metabolite produced by *Aspergillus* and *Penicillium*, including *A. carbonarius*, *A. niger*, *A. steynii*, *A. westerdijkiae* and *P. nordicum* [5,6]. Some of the major OTA-producing species belong to *Aspergillus* section *Circumdati,* and these include the highly morphologically similar *A. westerdijkiae*, *A. ochraceus* and *A. steynii* [6]; all of these are the most important species for possible OTA contamination in agricultural products [7]. As one of the important OTA-producing fungi, *A. westerdijkiae* can infect wheat, rice, beverages, salt, sorghum, corn, peppers, fennel, pear, grapes and many other foods and crops [8]. *A. westerdijkiae* exhibits vigorous growth in warm climates or during storage conditions. The production of OTA is significantly influenced by the specific substrate used. In particular, the host crops of the fungus such as barley and peppers are particularly conducive to high OTA productivity and pose a considerable risk of OTA contamination [9]. At the same time, *A. westerdijkiae* was considered a potential hazard for animal-derived products, such as salt-rich dry-cured ham [10].

Mitogen-activated protein kinase (MAPK) is a protein kinase found in eukaryotes [11]. MAP kinase controls many complex processes in cells, including embryogenesis, cell proliferation, differentiation and decay and is associated with homeostatic regulation of the cell cycle [12]. MAPK is a conserved evolutionary module consisting of three kinase cascades, MAPK, MAPK kinase (MAPKK) and MAPKK kinase (MAPKKK) [13,14,15]. Three cascades of kinases respond to environmental signals, and the cell responds in order to survive. In response to increased environmental stress, yeast initiates a series of adaptive responses, including the synthesis of compatible osmotic glycerol, altered patterns of gene expression and protein synthesis [14]. In fungal cells, the MAPK signaling pathway plays a key role in regulating development by transmitting extracellular signals to the nucleus after sensing stimuli from the surrounding environment [15]. The MAPK pathway is commonly associated with oxidative responses, osmoregulation and aspects of fungicide susceptibility in plant pathogens [16,17,18]. MAPKs, also known as SAPKs (stress-activated protein kinases), generate multiple stress responses through environmental signaling. The MAPK SakA is a component of a cascade stress signaling pathway [19] that can generate multiple stress responses.

The cascade pathway can influence mycotoxin formation by transducing environmental signals into biological signals. Regarding SakA MAPK, its involvement in mycotoxin synthesis has been demonstrated in *Aspergillus flavus* [11], *Fusarium verticillioides* [20] and *Penicillium marneffei* [21]. *A. nidulans* contained all genes homologous to the high-osmolarity glycerol response MAPK pathway of *Saccharomyces cerevisiae*, and knockout strains of the MAPK homologous protein exhibited growth inhibition at high osmolarity [22]. In *Alternaria alternata*, MAP kinase is responsible for converting the signals related to high osmolarity into cellular responses. Different levels of NaCl cause the MAP kinase to become phosphorylated (activated) in a manner that depends on the concentration of salt, the length of exposure to it and the specific strain of the fungus. This, in turn, affects the process of alternariol mycotoxin biosynthesis from fungi that grow on vegetables [23]. A pathogenicity test was conducted by Igbalajobi and his team on a strain with a MAPK Δ*hogA*-deletion. The strain showed significant colonization impairment of tomato and apple when conidia were used as the inoculum. However, when hyphae were used as inoculum, the fruit was colonized very effectively [24]. *A. fumigatus* MpkC and SakA are homologs of *S. cerevisiae* Hog1 and are involved in the regulation of oxidative stress, cell wall damage and other responses as part of the osmotic and general signaling pathways [25]. SakA is essential for the viability of conidia and survival at high H_2_O_2_ concentrations [26]. Different salt concentrations affected the growth rate, toxin production and phosphorylation in *Penicillium verrucosum,* and this was regulated by MAPK [27]. In *P. nordicum* and *P. verrucosum*, production of OTA induced by NaCl is associated with the phosphorylation of MAP kinase, whereas in *A. carbonarius* the opposite is true [17]. The osmotic adaptation response is conserved and is closely related to the morphogenesis in filamentous fungi but not identical in different fungi [28]. However, further research is still needed on how fungi use the MAPK pathway to regulate specific biological functions.

Although SakA has been investigated in certain fungi, its regulatory role in *A. westerdijkiae* remains unclear to our knowledge. Our aim was to determine the regulatory role of SakA for *A. westerdijkiae*. For this purpose, we identified and deleted *SakA* in *A. westerdijkiae* using an efficient genetically transformed strain and explored the effects of environmental factors on the growth and OTA production of *A. westerdijkiae* WT and *ΔAwSakA*. We demonstrate that osmotic stress and cell wall damage significantly affect growth and development and OTA biosynthesis in *A. westerdijkiae* and that *SakA* plays an important role in this process. A reduction in OTA production is associated with a downregulation of synthetic genes, and *SakA* affects the pathogenicity of *A. westerdijkiae* towards pear and grape. This paper aids important information and acts as a source for understanding the role of *SakA* under various stress conditions such as osmotic stress, cell wall damage and oxidative stress.

## 2. Results

### 2.1. Identification of SakA in A. westerdijkiae

**Figure 1:**
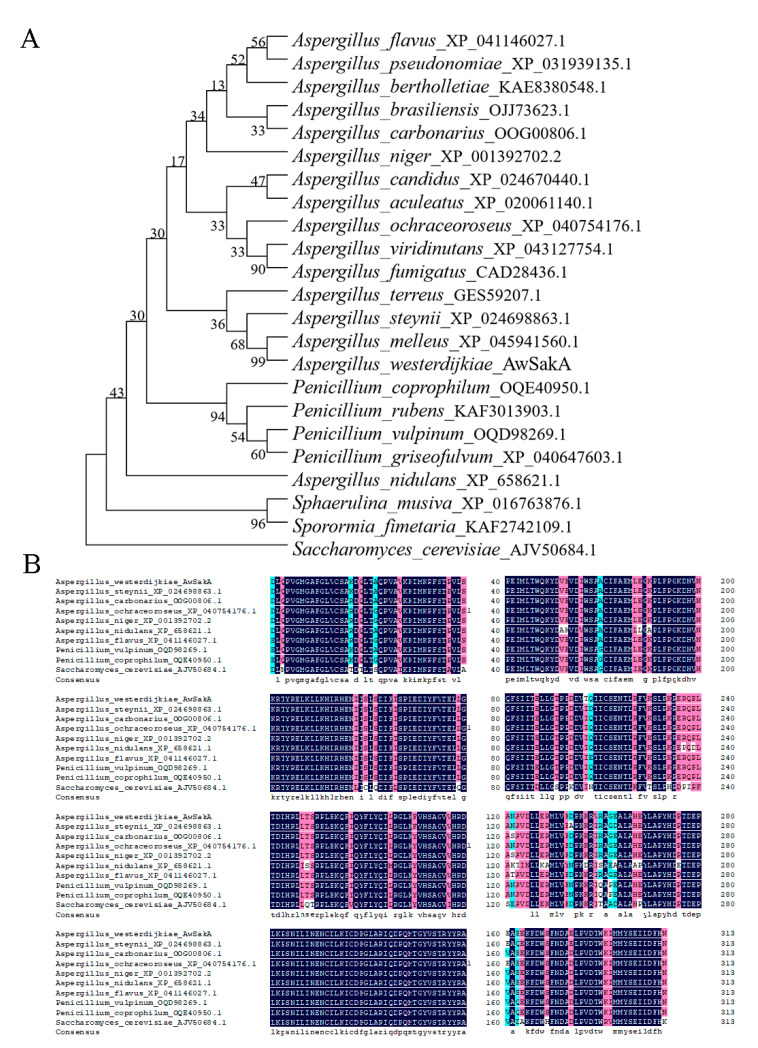
Phylogenetic relationships and domain analysis of AwSakA homologous proteins in different fungi. (**A**) Alignment of amino acids encoding AwSakA in *A. westerdijkiae* with the homologous proteins from 22 fungi. (**B**) Amino acid sequence alignment of protein kinase structural domains of homologues of AwSakA in *A. westerdijkiae*, *A. steynii*, *A. carbonarius*, *A. ochraceoroseus*, *A. niger*, *A. nidulans*, *A. flavus*, *P. vulpinum*, *P. coprophilum* and *S. cerevisiae*.

In order to identify homologues of the SakA protein in *A. westerdijkiae*, the protein sequences of SakA were homology matched using protein BLAST from the National Center for Biotechnology Information (NCBI). *AwSakA* encodes 314 amino acids in *A. westerdijkiae*. A total of 23 homologous proteins from fungal species were used to construct a phylogenetic tree (Figure 1A), including 16 species of *Aspergillus*, 4 species of *Penicillium* and *Saccharomyces* species, etc. As shown in Figure 1A, SakA from *A. melleus* (XP_045941560.1) was most related to AwSakA from *A. westerdijkiae*, with a similarilty of 97.52%. SakA from the genera *Aspergillus* and *Penicillium* exhibited a high degree of homology. Ten fungi (*A. westerdijkiae*, *A. steynii*, *A. carbonarius*, *A. ochraceoroseus*, *A. niger*, *A. nidulans*, *A. flavus*, *P. vulpinum*, *P. coprophilum* and *S. cerevisiae*) were selected for comparative amino acid sequence alignment analysis (Figure 1B). The amino acids encoding SakA were similar in length across the different fungal species, all species have a protein kinase encoded by 126 amino acids. Among these, a conserved protein kinase structural domain was found with the same binding site in different strains, which is particularly important for studying the function of *SakA*.

### 2.2. Generation of AwSakA Deletion Mutants

**Figure 2:**
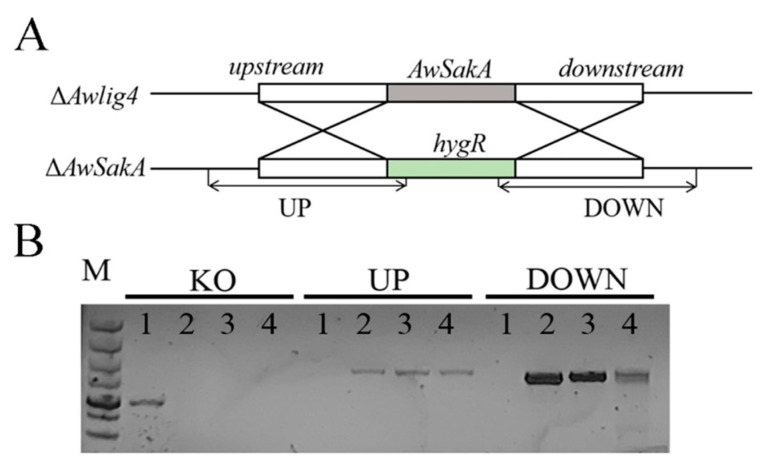
Development strategy of *A. westerdijkiae ΔAwSakA* strains. (**A**) Schematic diagram of *AwSakA* knockout strategy. (**B**) PCR validation of *A. westerdijkiae* WT and *ΔAwSakA*, WT (1) and three mutants *ΔAwSakA*-1 (2), *ΔAwSakA*-2 (3) and *ΔAwSakA*-3 (4) were identified by the paired primers of KO, UP and DOWN; M: 5kb maker.

*ΔAwlig*4, the non-homologous end-joining pathway of which was disrupted previously [29], was used as the WT strain to knock out *AwSakA*. *AwSakA* was replaced with *hygR* using a strategy of replacing the target gene by homologous recombination; the strategy of construction is shown in Figure 2A. Hygromycin B was added to potato dextrose agar (PDA) medium, *A. westerdijkiae* (WT) was unable to grow on plates with hygromycin B, whereas *ΔAwSakA* was able to grow normally due to integration with *hygR*. Eight mutant strains were screened. The genomic DNA of the transformants was extracted and identified by PCR amplified fragments UP and DOWN. Five mutants were eventually identified as positive transformants. In deletion mutants *ΔAwSakA-*1, *ΔAwSakA-*2 and *ΔAwSakA-*3, the homologous fragment containing *hygR* could be detected, whereas none were seen in *A. westerdijkiae* (Figure 2A). It was thus determined that *SakA* had been knocked out and that there was no ectopic insertion. The mutants *ΔAwSakA-*1, *ΔAwSakA-*2 and *ΔAwSakA-*3 were considered as three biological replicates of *A. westerdijkiae* in subsequent experiments.

### 2.3. Growth, Conidiation and Sclerotia Formation of A. westerdijkiae Were Modulated by AwSakA

**Figure 3:**
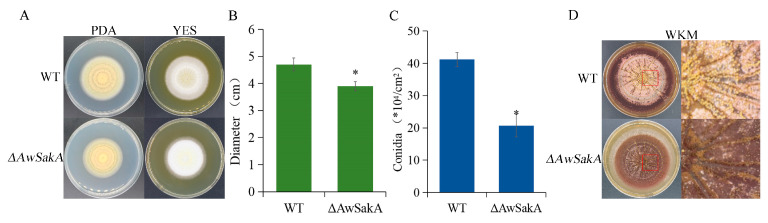
Effect of *AwSakA* deletion on the growth rate of *A. westerdijkiae*, sclerotia formation and germination of conidia. (**A**) Growth phenotype of WT and *ΔAwSakA* on PDA and YES for 7 days. (**B**) Growth diameter of WT and *ΔAwSakA* on YES for 7 days. (**C**) Conidial production of WT and *ΔAwSakA* at 5 days on PDA. (**D**) Sclerotia formation and enlarged portion of WT and *ΔAwSakA* after 11 days on WKM in magnified form. Both WT and *ΔAwSakA* had three biological replicates, and each strain was cultured on four plates as technical replicates. The asterisk indicates a significant decrease between the corresponding values (*p* < 0.05).

To study the effect of *AwSakA* deletion on the growth and conidia production of *A. westerdijkiae*, WT and *ΔAwSakA* were inoculated on yeast extract sucrose (YES) and PDA. As shown in Figure 3A, *AwSakA* deletion retarded the growth rate of *A. westerdijkiae*, and the results were confirmed by the colony diameters (Figure 3B). At the same time, conidiation of WT and *ΔAwSakA* on PDA was tested and the results are shown in Figure 3C; compared with WT, conidial production decreased by approximately 47% due to the deletion of *AwSakA*. In addition, after 11 days of cultivate in WKM solid medium, numerous sclerotia were present in WT but none were in *ΔAwSakA*, as shown in Figure 3D.

### 2.4. The Effect of Environmental Stress Agents on Growth and Conidial Production of A. westerdijkiae Are Regulated by AwSakA

**Figure 4:**
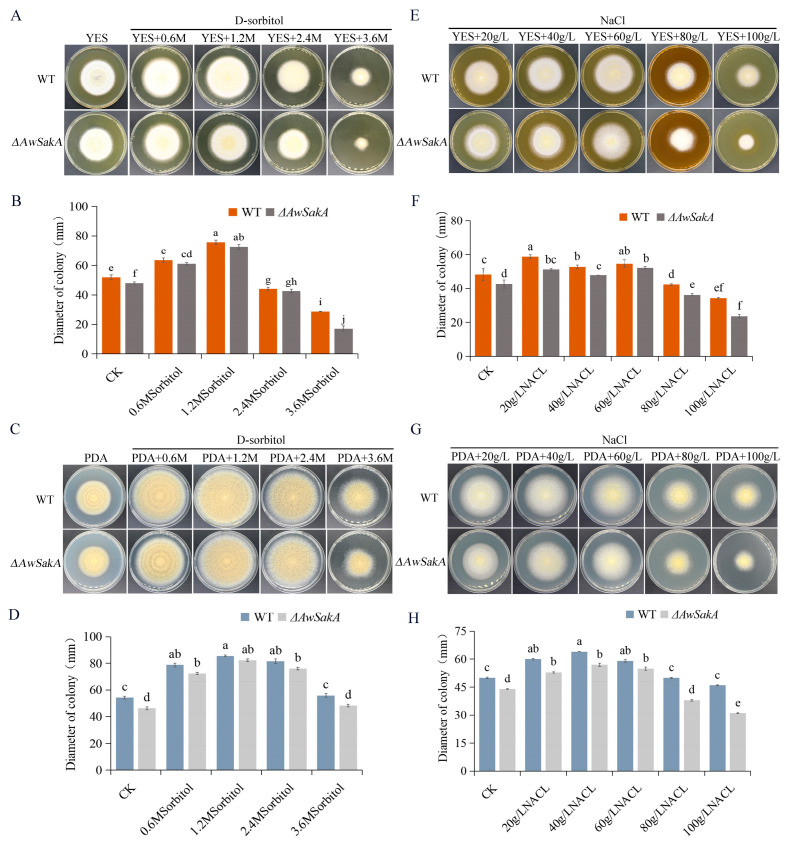
Effects of osmotic stress on growth of *A. westerdijkiae* and *ΔAwSakA* at different concentrations. (**A**) D-sorbitol treatment on YES. (**B**) Colony diameter on YES after 7 days of treatment with D-sorbitol. (**C**) D-sorbitol treatment on PDA. (**D**) Colony diameter on PDA after 9 days of treatment with D-sorbitol. (**E**) NaCl treatment on YES. (**F**) Colony diameter on YES for 7 days of treatment with NaCl. (**G**) NaCl treatment on PDA. (**H**) Colony diameter on PDA for 9 days of treatment with NaCl. Each treatment had three biological replicates, and each strain was cultured on four plates as technical replicates. Different letters indicate a significant difference between the corresponding values (*p* < 0.05).

**Figure 5:**
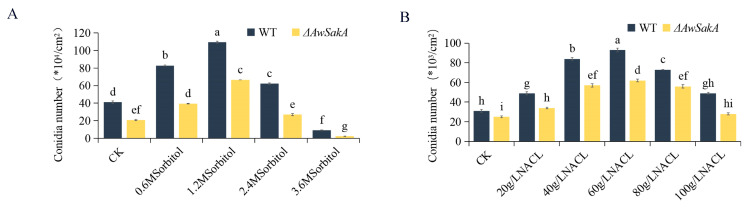
Effects of osmotic stress on conidiospore production of *A. westerdijkiae* and *ΔAwSakA* at different concentrations. (**A**) Production of conidia on PDA by WT and *ΔAwSakA* treated with D-sorbitol. (**B**) Production of conidia on PDA by WT and *ΔAwSakA* treated with NaCl. Each treatment had three biological replicates, and each strain was cultured on four plates as technical replicates. Different letters indicate a significant difference between the corresponding values (*p* < 0.05).

Different concentrations of D-sorbitol (0.6 M, 1.2 M, 2.4 M, 3.6 M) and NaCl (20 g/L, 40 g/L, 60 g/L, 80 g/L, 100 g/L) were selected to study the effects of different osmotic stress on growth and conidial production. The growth of *A. westerdijkiae* showed a concentration dependence for different osmotic stress. Appropriate concentrations of osmotic agents promote the growth of *A. westerdijkiae*, with concentrations of 1.2 M D-sorbitol (Figure 4A,C) and 60 g/L NaCl (Figure 4E,G) showing relatively high growth and conidial production. However, when the concentration increases from this limit, the growth and conidial production of *A. westerdijkiae* and *ΔAwSakA* were inhibited to varying degrees. As shown in Figure 4B, treatment of 1.2 M D-sorbitol on YES medium increased the diameters of WT and *ΔawSakA* by 45.5% and 50.9%, respectively. When the concentration of D-sorbitol was 3.6 M, the inhibition effect was more noticeable in *ΔAwSakA* than in WT. At this concentration, the size of WT and *ΔAwSakA* decreased by 44.5% and 64.2%, respectively. In contrast, on PDA medium, D-sorbitol promoted mycelial growth at concentrations 0.6 M and 1.2 M, where WT and *ΔAwSakA* diameters increased by 51% and 64%, respectively, but growth was inhibited at 3.6 M (Figure 4C,D). As shown in Figure 4F, the growth inhibition rates of WT and *ΔAwSakA* by 100 g/L NaCl on YES medium were 28.9% and 44.5%, respectively. In contrast, the growth inhibition rates of *ΔAwSakA* were 14.6% and 29.6% on PDA medium at 80 g/L and 100 g/L NaCl, respectively (Figure 4G,H). Under 3.6 M D-sorbitol, conidial production of WT and *ΔAwSakA* showed significant inhibitions (Figure 5A). As a result of NaCl treatment, the germination of conidia declined after 60 g/L, whereas 100 g/L NaCl treatment also inhibits the growth of hyphae, as compared with CK where the production of conidia is not inhibited (Figure 5B). It is indicated that osmotic stress as an environmental factor can affect the growth of *A. westerdijkiae* and that this action is dependent on the type of osmotic stress and controlled by *AwSakA*.

**Figure 6:**
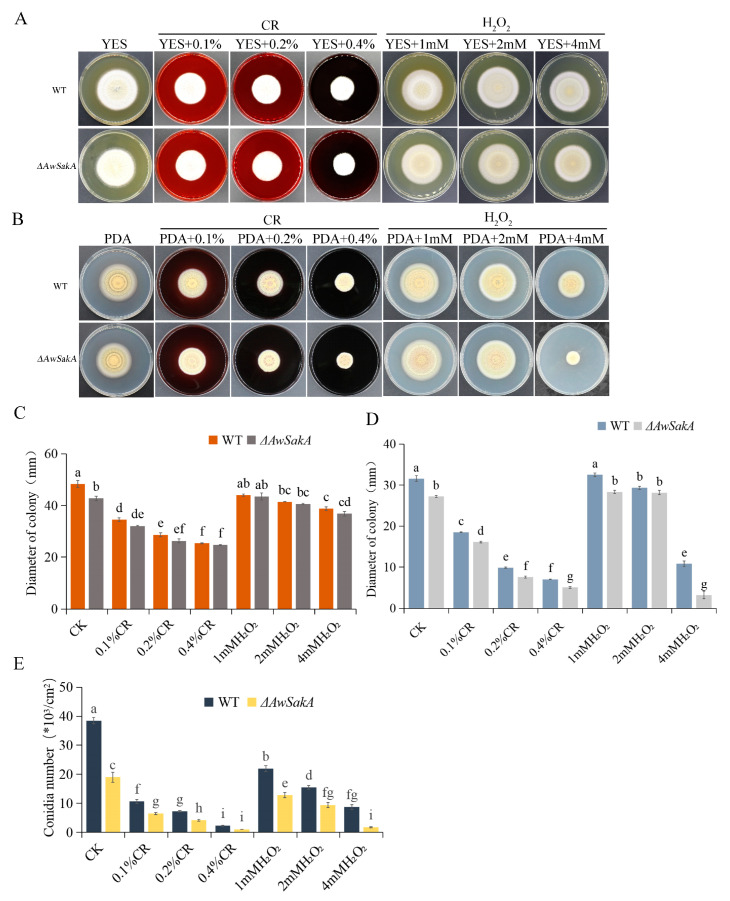
Effects of coercive agent stress on growth and conidiospore production of *A. westerdijkiae* and *ΔAwSakA* at different concentrations. (**A**) Growth phenotypes of WT and *ΔAwSakA* on YES treated with Congo red and H_2_O_2_. (**B**) Growth phenotypes of WT and *ΔAwSakA* on PDA treated with Congo red and H_2_O_2_. (**C**) Colony diameter of WT and *ΔAwSakA* on YES after 7 days of treatment with Congo red and H_2_O_2_. (**D**) Colony diameter of WT and *ΔAwSakA* on PDA after 7 days of treatment with Congo red and H_2_O_2_. (**E**) Production of conidia on PDA by WT and *ΔAwSakA* under Congo red and H_2_O_2_ treatment. Each treatment had three biological replicates, and each strain was cultured on four plates as technical replicates. Different letters indicate a significant difference between the corresponding values (*p* < 0.05).

Different concentrations of Congo red and H_2_O_2_ were added to YES and PDA media to investigate the response of *A. westerdijkiae* and *ΔAwSakA* to cell damage and oxidative stress. Growth phenotypes, asexual development and conidiation were observed in WT and *ΔAwSakA* strains of *A. westerdijkiae*. With addition of Congo red at final concentrations of 0.1%, 0.2% and 0.4%, WT and *ΔAwSakA* had similar growth morphology on YES, as shown in Figure 6A. With folds being produced on the surface of the colonies, the WT has more pronounced folds than *ΔAwSakA*. Both strains displayed an inhibitory effect on the growth of the colonies as the concentration of Congo red increased; the inhibition of WT and *ΔAwSakA* by a concentration of 0.4% Congo red was 69.8% and 68.9%, respectively, (Figure 6C). Different volumes of H_2_O_2_ with final concentrations of 1 mM, 2 mM and 4 mM were added; the effect of H_2_O_2_ is more distinct in PDA than YES. The growth of hyphae is hardly affected by the concentration of H_2_O_2_ on YES media. Growth inhibition of WT and *ΔAwSakA* on PDA was best achieved by 4 mM H_2_O_2_ (Figure 6A,B). The colony diameters on PDA medium are shown in Figure 4D. The addition of Congo red inhibited the mycelial growth of *A. westerdijkiae*, and the absence of *AwSakA* made the fungus more sensitive to this treatment. It is noteworthy that the growth of *ΔAwSakA* was significantly inhibited compared with WT when treated with 4 mM H_2_O_2_. As shown in Figure 6E, the addition of both Congo red and H_2_O_2_ inhibited the conidial production of WT and *ΔAwSakA* on PDA medium with a concentration dependence trend.

### 2.5. AwSakA Was Involved in the Biosynthesis of OTA

**Figure 7:**
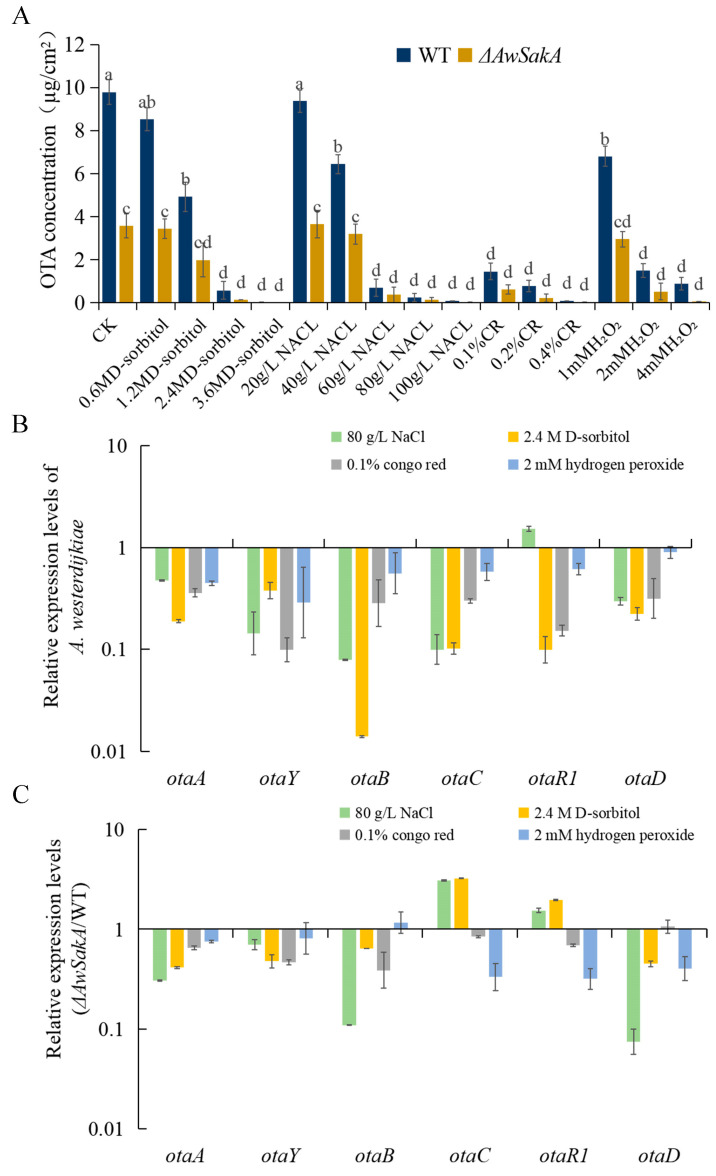
The effect of different types of coercive agents on OTA biosynthesis in *A. westerdijkiae* WT and *ΔAwSakA*. (**A**) Production of OTA in WT and *ΔAwSakA* strains of *A. westerdijkiae* by D-sorbitol, NaCl, Congo red and H_2_O_2_. Different letters indicate significant differences between the corresponding values (*p* < 0.05) with three biological replicates. (**B**) Relative expression level of OTA biosynthetic genes of WT on media containing D-sorbitol, NaCl, Congo red and H_2_O_2_ analyzed by RT-qPCR. (**C**) The expression ratio (*ΔAwSakA*/WT) of OTA biosynthetic genes on media containing D-sorbitol, NaCl, Congo red and H_2_O_2_. Each treatment represents the mean of three independent experiments and the standard error is indicated by the bars.

To investigate whether *AwSakA* is involved in the biosynthesis of toxin, OTA was extracted from YES using different coercive chemical agents and its production was determined by HPLC. The production of OTA was significantly reduced in *ΔAwSakA* compared with WT, with a reduction rate of approximately 54%. Different concentrations of D-sorbitol, NaCl, Congo red and H_2_O_2_ treatments cause variations in the production of OTA. *A. westerdijkiae* can produce high levels of OTA at lower concentrations of osmotically active compounds, whereas OTA production was decreased in the presence of higher concentrations of compounds. In our study, D-sorbitol concentrations above 1.2 M and NaCl above 40 g/L became a sensitivity threshold level for the osmolality signal. OTA production was undetectable at 3.6 M sorbitol concentrations. In both WT and *ΔAwSakA* strains, the production of OTA is affected by the concentration of NaCl, following a similar trend to that of D-sorbitol treatment. Previous studies have reported that *A. westerdijkiae* grew well in salty environments [10], but when the concentration of NaCl exceeded 40 grams per liter, the production of OTA decreased. At a very high concentration of salt (100 grams per liter of NaCl), only very small amounts of OTA (90 ng/cm^2^ in WT and 13 ng/cm^2^ in *ΔAwSakA*) were detected. It was observed that even a small amount of 0.1% Congo red could significantly reduce the production of OTA. When Congo red was added to the YES medium, it inhibited the production of OTA in both *A. westerdijkiae* WT and *ΔAwSakA*. The addition of 0.1%, 0.2% and 0.4% Congo red resulted in 80%, 82% and 87% reductions, respectively, in OTA production in *ΔAwSakA*. *A. westerdijkiae* responded to oxidative stress compared with the untreated group; 1mM H_2_O_2_ decreased the production of OTA from 9.79 ± 0.59 µg/cm^2^ to 6.80 ± 0.74 µg/cm^2^, and the production of OTA decreased with the elevated H_2_O_2_ in *A. westerdijkiae* WT and *ΔAwSakA*. At 4 mM of H_2_O_2_, the production of OTA in *A. westerdijkiae* WT and *ΔAwSakA* decreased by 74.6% and 82.4%, respectively (Figure 7A). It is obvious that OTA production in the deletion of *AwSakA* was significantly lower than that in WT under the same treatment. The production of OTA in *A. westerdijkiae* is influenced by environmental signals, and *AwSakA* responds to various stimuli such as high osmolarity, oxidative stress, and cell damage, all of which can affect OTA biosynthesis. These effects are dependent on the concentration of the stimuli.

As previously described, concentrations of coercive chemical agents significantly inhibited OTA toxicity in *A. westerdijkiae*. Therefore, to check the gene expression of the OTA regulatory genes, we isolated RNA from *A. westerdijkiae* WT and *ΔAwSakA* under four different stresses (2.4 M D-sorbitol, 80 g/L NaCl, 0.1% Congo red and 2 mM H_2_O_2_) from cultured the strains in solid YES for 9 days and analyzed the expression levels of six biosynthetic genes for OTA, including *otaA*, *otaY*, *otaB*, *otaC*, *otaD* and *otaR*1. As shown in Figure 7B, the relative expression levels of all six genes were downregulated when *A. westerdijkiae* was cultured on media containing 2.4 M D-sorbitol, 0.1% Congo red and 2 mM H_2_O_2_ compared with the absence of environmental stress, whereas the upregulation of transcription factor *otaR*1 in NaCl was not significant. In addition, the relative expression of genes in *ΔAwSakA* was compared with that in *A. westerdijkiae* WT. Knockout of *AwSakA* regulated the expression of OTA biosynthetic genes, with the expression of *otaA*, *otaY*, *otaB* and *otaD* showing downregulation in NaCl and D-sorbitol treatment; all genes in Congo red treatment and all genes except *otaB* in H_2_O_2_ treatment were downregulated (Figure 7C). Taken together, these results indicate that specific concentrations of environmental stress and knockout of *AwSakA* resulted in a downregulation in the OTA synthesis cluster. *AwSakA* regulates the expression of biosynthetic genes and has a positive regulatory effect on OTA production in *A. westerdijkiae*.

### 2.6. The Role of AwSakA in the Pathogenicity of A. westerdijkiae

**Figure 8:**
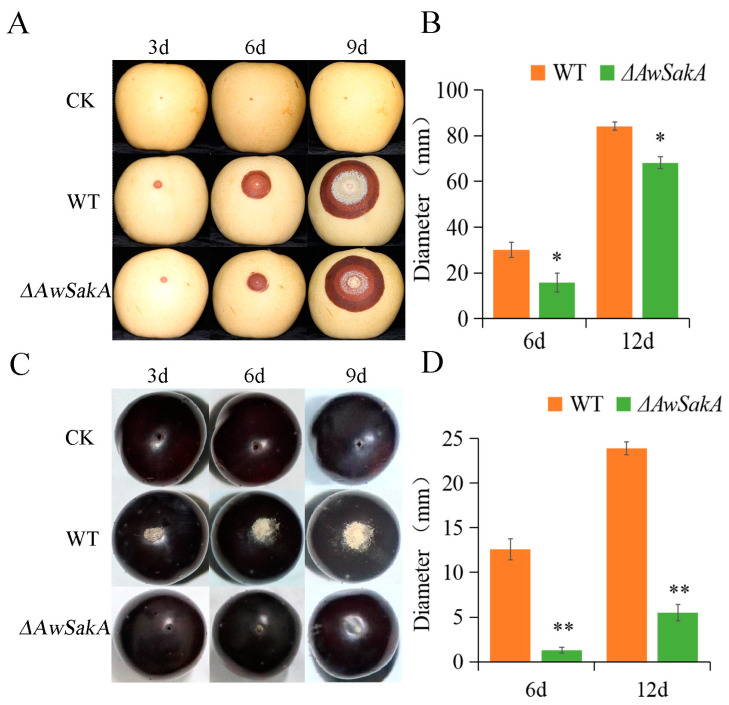
Pathogenicity assay for WT and *ΔAwSakA* of *A. westerdijkiae* on pears and grapes. (**A**) Infestation of *A. westerdijkiae* WT and *ΔAwSakA* on pears. (**B**) Diameter of lesion on pears. (**C**) Infestation of grapes by *A. westerdijkiae* WT and *ΔAwSakA*. (**D**) Diameter of lesion on grapes. The asterisks indicate a significant decrease between the corresponding values (* *p* ≤ 0.05 and ** *p* ≤ 0.01) with three biological replicates.

To investigate the role of *AwSakA* in pathogenicity, crown pears and Kyoho grapes were selected as hosts for infection experiments and phenotypes were recorded at 3, 6 and 9 days after inoculation. The diameters of lesions on days 6 and 12 were counted. It was observed that the lesions of *A. westerdijkiae* were larger than *ΔAwSakA* and there were more conidiospores on the surface of pears (Figure 8A,B). In Kyoho grapes, *ΔAwSakA* produced a smaller number of conidiospores and was less virulent than the *A. westerdijkiae* WT (Figure 8C,D). As the duration of infection persisted, the lesions of *A. westerdijkiae* progressively deepened and those of *ΔAwSakA* were more slowed.

## 3. Discussion

OTA is a toxic secondary metabolite produced by some *Aspergillus* and *Penicillium* species. The biosynthesis and regulatory mechanisms of OTA have been well studied in OTA-producing fungi. *A. westerdijkiae* is one of the common OTA-producing fungi, causing serious contamination of food and foodstuffs; however, the biosynthesis of OTA in *A. westerdijkiae* is insufficiently studied, especially the regulatory role of *SakA*.

Phosphorophore system-coupled MAPKs (mitogen-activated protein kinases) in fungi sense and process environmental signals [30]. MAP kinases transmit signals generated at the cell surface or in the cytoplasm to the nucleus and regulate gene expression [31]. It has been shown that the MAPK signaling pathway plays an important role in the growth and development of eukaryotes [7]. MAPKs play a crucial role in various cellular processes within eukaryotes, and there are different MAPK signaling pathways in fungi, including SakA-MAPK, Slt2-MAPK and Fus3-MAPK [32]. In *S. cerevisiae*, SakA acts as an upstream receptor for Snf1 and is involved in crosstalk between the energy-sensing signaling pathway and Snf1 [33]. In *A. fumigatus*, the homologs of *Hog*1 from *S. cerevisiae*, namely *MpkC* and *SakA*, are significant in adaptation to oxidative and osmotic stresses, cell wall damage, macrophage recognition, heat shock and virulence [25,34]; there has been evidence of an interaction between SakA and cell-wall-stress pathways [35]. MAPK genes are involved in various pathways; studies have defined the specific functions of each pathway, such as MAPK in fungal physiology, the HOG pathway’s role in stress sensing [36], Mkc1 in cell wall integrity [34], Cek1 in the invasion and synthesis of an essential cell wall component [37] and Cek2 in pheromone receptors or mating [38]. However, further research has shown that most MAPK pathways in fungi play a multidimensional role due to their connections and crosstalk with other pathways in the cell. As part of MAPK family, *SakA* can transmit osmotic and oxidative signals [39]. As a regulatory factor in response to osmosis in fungi, *SakA* is extremely important for the synthesis and regulation of OTA and acts as a homologue of *hog*1 in *S. cerevisiae* in response to multiple stress responses [40].

In this study, we focus on the role of *AwSakA* in regulating morphogenesis, conidia production, OTA synthesis and the pathogenicity of *A. westerdijkiae*, how *AwSakA* responds to osmotic stress signals and the effects of different stressors on OTA biosynthesis. Molecular biological studies of gene function in OTA-producing fungi are commonly identified by homologous knockout systems. There are disadvantages such as low transformation efficiency and many false positives. We used *ΔAwlig*4 [29], which broke the non-homologous end-joining pathway, for the construction of *ΔAwSakA* mutants, which greatly improved the efficiency of gene targeting. During the identification process of *AwSakA*, we found that *SakA* is evolutionarily conserved in the *Aspergillus*, and a comparative sequence analysis of all fungal species showed that *SakA* is highly similar and that it shares the same highly conserved protein kinase structural domain. This is consistent with the study showing that *SakA* encodes part of the MAPK gene family in the fungi *A. alternata* [16] and *P. verrucosum* [18]. We identified the gene function of *AwSakA* and investigated the growth and conidial and OTA production of *ΔAwSakA*. *AwSakA* regulated the growth of *A. westerdijkiae*, affected sclerotia germination and was a positive regulator of OTA production. The data showed that the growth of *ΔAwSakA* was inhibited compared with wild-type colony growth. On the other hand, in comparison to the WT, the deletion of *AwSakA* resulted in a significant decrease in conidial production. Moreover, the absence of *AwSakA* led to the absence of sclerotia in *A. westerdijkiae*, as compared with the numerous sclerotia in the WT. It was hypothesized that *ΔAwSakA* may have a negative impact on OTA production, and this was confirmed by this study, which showed that the *SakA* mutant caused low OTA production.

As in previous studies, *ΔSakA* mutants showed reduced trehalose content, were sensitive to heat, osmotic, and oxidative stresses and were also sensitive to cell wall damaging agents, leading to reduced spore viability in *A. fumigatus* [37]. Therefore, these earlier studies support our results. In parallel to this study, an earlier study relating to this was conducted by Ochiai, N. et al., were they studied mutant strains of *Fusarium graminearum* lacking MAPKKK encoding genes, such as *FgOs*4, *FgOs*5 (MAPKK) and *FgOs*2 (MAPK), and were unable to produce trichothecenes mycotoxins in aerial hyphae [41].

Furthermore, we investigated the phenotype and toxicity production of WT and *ΔAwSakA* strains of *A. westerdijkiae* under different environmental stresses. WT and *ΔAwSakA* showed different abilities for growth, conidial production and OTA biosynthesis; deletion of *AwSakA* made the strain more sensitive to stress. Low osmolarity/osmotic stress (D-sorbitol, NaCl) promotes the colony growth and conidial germination to some extent, whereas a high concentration of osmotic stress significantly inhibits both colony growth and conidia at higher levels. Transcriptional regulation of osmotic stress is dependent on SakA and MpkC [42]. After comparing these results with previous studies, we discovered that in *A. fumigatus ΔSakA* gene mutants, the cell wall integrity pathway was not activated. This led to these mutants being more sensitive to agents that damage the cell wall [43]. The synthesis of sphingolipids (SL) in yeast is regulated by the AGC kinases Ypk1 and Ypk2; SL synthesis is associated with the cell wall integrity (CWI) and the high osmolarity glycerol (HOG) pathways, and in *A. fumigatus*, *YpkA* physically interacts with *SakA* MAP kinase to regulate SL biosynthesis [44]. MAPK pathway genes are known to interact with and regulate other signaling pathways in the cell, forming complex networks of signaling cascades [45]. For example, MAPK pathway genes can interact with the *PI*3*K/Akt* pathway within cells that are activated by external signals and this stimulates metabolic processes, cell growth, proliferation, survival and angiogenesis [46,47]. Therefore, mutation or silencing of this gene may disturb these mechanisms. The germination of conidia decreased after 60 g/L NaCl treatment, and higher concentrations inhibited the growth of hyphae as well. In comparison with the control group, where conidia production was not hindered, these results suggest that osmotic stress can impact the growth of *A. westerdijkiae*. The fungal strain became more sensitive to osmotic stress in the absence of *AwSakA*. Moreover, increasing concentrations of Congo red had an inhibitory effect on the growth of colonies for both WT and mutant strains. The growth inhibition effect of H_2_O_2_ was more noticeable in PDA than YES media for hyphae. The study findings demonstrate that the addition of both Congo red and H_2_O_2_ led to inhibition of conidial production in WT and Δ*AwSakA* strains, with a trend that was dependent on the concentration. In addition, compared with the WT, the absence of *AwSakA* resulted in the downregulation of *otaA*, *otaY*, *otaB* and *otaD* under treatment with 80 g/L NaCl and 2.4 M D-sorbitol, with NaCl treatment having a more pronounced effect on this downregulation. In contrast, 0.1% Congo red and 2 mM H_2_O_2_ downregulated the transcript levels of five genes in the OTA synthesis cluster, although this regulation was not significant. In addition, all four treatments at all concentrations significantly inhibited OTA biosynthesis. Similar to *A. flavus*, the *AfSakA* gene is found to be involved in responses to hyperosmotic stress due to NaCl and D-sorbitol as the *ΔAfSakA* mutant was more sensitive to hyperosmotic stress [11]. Exposure of bacteria, particularly *Escherichia coli* and *Salmonella typhimurium*, to hydrogen peroxide leads to the activation of the transcription factor OxyR, which regulates the expression of nine genes required for protection against peroxidative stress, including catalase and glutathione reductase [48]. We explored the regulatory role of Congo red and H_2_O_2_ and found that *ΔAwSakA* and the WT were sensitive to stress in response to low concentrations of cell wall damage. Consistent with the results in *A. fumigatus*, adding H_2_O_2_ to the medium increased the growth inhibition of the *SakA* mutant and without oxidative stress *SakA* was required for the optimal growth of the organism [40]. Similar to the effects caused by osmolality, the absence of *AwSakA* was more sensitive to the responses of different concentrations H_2_O_2_ compared with *A. westerdijkiae.*

For virulence or pathogenicity tests, it was noticed that the lesions caused by *A. westerdijkiae* were significantly larger than those caused by *ΔAwSakA* on the surface of pears. In the case of Kyoho grape infections, the number of conidia and the diameter of lesions caused by *A. westerdijkiae* (WT) were considerably greater than in *ΔAwSakA*. Moreover, as the infection persisted, the lesions caused by *A. westerdijkiae* became rapidly deeper, whereas those caused by Δ*AwSakA* showed slower progression. One of the previous studies showed that *A. ochraceus* conidiospores on oats infected by the WT increased over time, whereas in oats that were infected by *ΔAoCreA*, the conidiospore numbers were significantly lower. On the other hand, pears infected with the WT showed a rapid increase in lesions, whereas the lesions in *ΔAoCreA* were small and they were not rapidly expanding [49].

## 4. Conclusions

In conclusion, the *AwSakA* gene was deleted to obtain *ΔAwSakA* using a homologous recombination strategy in *A. westerdijkiae*. *AwSakA* has an important role in regulating morphogenesis, sclerotia production and phloem development in *A. westerdijkiae*. In terms of virulence, the knockout of *AwSakA* inhibited toxin synthesis by downregulating the expression of OTA synthesis genes and delayed the pathogenicity of *A. westerdijkiae* to agricultural products by inhibiting the germination of conidia. In addition, the effects of different environmental stresses on the growth of WT and *ΔAwSakA* were presented; *ΔAwSakA* showed a quite interesting response in the regulation of osmotic stress, ionic stress and cell wall integrity in *A. westerdijkiae*. Based on a thorough understanding of the *AwSakA* gene’s actions, it is thought that this research offers trustworthy and useful information about the role of *AwSakA* in the morphological development and OTA biosynthesis in *A. westerdijkiae*. Our findings also provide important data that aid in our understanding of how to manage *A. westerdijkiae* infections and how to prevent the production of OTA toxicity.

## 5. Materials and Methods

### 5.1. Strains and Culture Conditions

The *A. westerdijkiae* used in the experiments was provided by our laboratory and stored at −80 °C [5]. The whole genome was previously sequenced [50]. Strains were activated with PDA medium incubated at 28 °C. In this study, Potato Dextrose Agar (PDA, Becton, Dickinson and Company, Franklin Lakes, NJ, USA) or Yeast Extract Sucrose (YES, yeast extract 20 g/L; agar 15 g/L; sucrose 150 g/L) was added with different concentrations of D-sorbitol, NaCl, Congo red or H_2_O_2_ and inoculated with the WT and gene deletion mutants to observe growth phenotypes and analyze secondary metabolites. Reagents were obtained from the supplier of Solarbio (Beijing, China) except for special markings. The infestation experiments of agricultural products were performed according to the conventional treatment method [49].

### 5.2. AwSakA Identification and Phylogenetic Analysis

The SakA amino acid sequences from *S. cerevisiae* was used as a query to find AwSakA using the Basic Local Alignment Search Tool (BLAST, NCBI, Bethesda, MD, USA), and other SakA sequences were downloaded from NCBI. ClustaIW was used to compare the amino acids of SakA, and a phylogenetic tree for SakA was constructed using Molecular Evolutionary Genetic Analysis Version 6.0 (MEGA 6.0) software and 1000 bootstrap replicates. Evolutionary genetic phylogenetic relationships and structural domains were aligned using DNAMAN (Lynnon Biosoft, Omega, San Ramon, CA, USA).

### 5.3. Acquisition of SakA Nucleic Acid Sequences and Primer Design

The whole genome of *A. westerdijkiae* was sequenced and uploaded to the NCBI with the accession No. of GCA_004849945.1. The genetic information for *AwSakA* was queried and downloaded from the NCBI database. Primers were designed according to SnapGene 3.2.1(GSL Biotech LLC, Boston, MA, USA), and the specificity of the designed primers was detected by NCBI. The primers used in this study are listed in Table 1.

### 5.4. Construction of Gene Deletion Mutant Strains

Construction of *A. westerdijkiae AwSakA* deletion mutants was performed using PCR procedures based on the principle of homologous recombination. The whole *AwSakA* gene was replaced by *hygR* (hygromycin B phosphotransferase). We selected base sequences greater than 1000 bp in length upstream and downstream of *AwSakA* for the homologous fragments of the recombination. Fusion of the three fragments was performed using double-joint polymerase chain reaction (DJ-PCR) procedures [51]. We used an efficient genetically transformed strain, *ΔAwlig*4 [29], to prepare protoplasts. Mutants were obtained by PEG-mediated protoplast transformation, and positive transformants were validated by diagnostic PCR [52].

### 5.5. Growth Phenotype Analysis

Different concentrations of D-sorbitol (0.6 M, 1.2 M, 2.4 M, 3.6 M), NaCl (20 g/L, 40 g/L, 60 g/L, 80 g/L, 100 g/L), Congo red (0.1%, 0.2%, 0.4%) and H_2_O_2_ (1 mM, 2 mM, 4 mM) were added to investigate the effect of *AwSakA* on nutritional growth. As Congo red is a carcinogen, it is recommended to follow standard laboratory safety protocols such as wearing gloves, lab coat and goggles and working in a well-ventilated area. The colonies were incubated at 28 °C; each colony was measured at right angles in horizontal and vertical directions and the diagonal of the right angle was used as a baseline to measure the diameter in both directions. We measured the diameter at four different angles using Vernier calipers (Mitutoyo, Kawasaki, Japan) and calculated the mean diameter. For the conidiation study, each plate was washed with 0.1% Tween-80 from PDA and counted using a hemocytometer (Marienfeld, Berlin, Germany). We adjusted the concentration of the conidial suspension prepared for inoculation to 10^7^ cfu/mL. A total of 3 µL of conidial suspension were inoculated in different concentrations of media and incubated at 28 °C in the dark. Each treatment had three biological replicates, and each strain was cultured on four plates as technical replicates.

### 5.6. OTA Production Analysis

*A. westerdijkiae* WT and *ΔAwSakA* were incubated for 10 days in different concentrations of coercive agent media and the whole plates were extracted for OTA production analysis. After extraction of the OTA by ultrasonication with ethyl acetate, the extract was concentrated by rotary evaporator (Yarong, Shanghai, China), resolved with 1 mL of chromatographic grade methanol, detection by Agilent HPLC system (Agilent Technologies, Santa Clara, CA, USA). The column was an Agilent ZorbaxSB-C18 (5 μm, 4.6 nm × 250 nm), the temperature of the column was set to 30 °C, the mobile phase was acetonitrile, water and acetic acid, the volume ratio was 99/99/2, the flow rate was 1.0 mL/min, 20 μL of extracts were used for analysis per test, the excitation wavelength of the fluorescence detector was 333 nm, the emission wavelength was 460 nm and each sample was run for 20 min [5]. The OTA content was calculated from the total OTA and the colony area. Reagents of ethyl acetate, methanol, acetonitrile and acetic acid were obtained from the supplier of Thermo Fisher (Waltham, MA, USA).

### 5.7. Extraction of RNA and preparation of cDNA

Mycelia of *A. westerdijkiae* were collected from YES solid medium incubated at 28 °C for 9 days and ground to a powder using a mortar and pestle in liquid nitrogen. Approximately 100 mg of the locked powder was used for RNA extraction. RNA was extracted using the Total RNA Extraction Kit (TianGen, Beijing, China) following the manufacturer’s protocol. RNA samples were measured using a Beckman DU800 (Beckman, Indianapolis, IN, USA). RNA purity was determined using the ratio of A260/A280 and agarose gel electrophoresis (180 v, 10 min). RNA was converted to cDNA using EasyScript^®^ One-Step gDNA removal and cDNA Synthesis SuperMix (TransGen, Beijing, China) according to the manufacturer’s protocol.

### 5.8. Quantitative Real-Time Polymerase Chain Reaction Analysis

We adjusted the concentration of the cDNA template to 100 ng/µL using a trace nucleic acid analysis and prepared the reaction system according to the Power SYBR Green MasterMix Kit (Takara, Kyoto, Japan). The *A. westerdijkiae GADPH* gene was used as an internal standard [5]. Gene expression was analyzed using an ABI 7500 real-time quantitative PCR system (Thermo Fisher, Waltham, MA, USA). The amplification primers used for RT-PCR are shown in Table 2; the relative expression tests for each gene were repeated three times. Data were processed according to the ABI 7500 v2.0.6 software (Thermo Fisher, Waltham, MA, USA), and relative expression of genes was calculated by the 2^−ΔΔCt^ method.

### 5.9. Pathogenicity Assay

Kyoho grapes and crown pears were selected as infestation hosts to study the pathogenicity of *A. westerdijkiae* WT and *ΔAwSakA*. Grapes on the same bunch were cut off with the stalk, the surface was cleaned and disinfected with 75% anhydrous ethanol, a wound about 2 mm deep was punctured on the top of the grapes with a sterile toothpick and 1 μL conidial suspension (10^7^ conidia/mL) was inoculated in the wound. For the treatment of the pears, the surface of the pear was also cleaned and disinfected with 75% anhydrous ethanol, a hole about 2 mm deep was punctured on the opposite side of the pear with a sterile toothpick and 1 μL conidial suspension was inoculated in the wound of the same pear. In the control group, 1 μL sterile water was inoculated. Both treatment and control groups were placed in a dark environment at 28 °C and incubated. We observed the development of lesions on the surface of the samples on days 3, 6 and 9. Each lesion was measured at right angles in horizontal and vertical directions, and the diagonal of the right angle was used as a baseline to measure the diameter in both directions. We measured the diameters at four different angles using Vernier calipers and calculated the mean diameter. Each experiment was set up with at least three biological replicates.

### 5.10. Statistical Analysis

Data were analyzed with IBM SPSS statistics version 25 and expressed as means and standard deviations. The mean values were compared by the least significant difference (LSD) and Duncan’s tests. Values were considered statistically significant when *p* < 0.05.

## Figures and Tables

**Table 1:** Primers for the construction of *A. westerdijkiae* knockout mutants.

Primers	Sequence (5′ to 3′)
*SakA*-up-F	CAGCCCAGATTGTATCTGAC
*SakA*-up-R	GCATTGATGTGTTGACCTCCCAGCCATACCAGACCAGTCC
*SakA*-down-F	CGAGGGCAAAGGAATAGAGTAGACGATGCTGAGCTACCAGTG
*SakA*-down-R	AGATCGCGTCAATAACTCCG
*SakA*-knock-F	TTAGGCATCGTGGGCTGACAC
*SakA*-knock-R	AACTTCTCTTCCGCCTCAGG
*hygR*-F	GGAGGTCAACACATCAATGCCTATTTTGGT
*hygR*-R	CTACTCTATTCCTTTGCCCTCGGACGAGTG
N-*SakA*-F	AGGAACATCTTGCATGGGCA
N-*SakA*-R	TCCTGGTGATACGGAGGGAG
C-*SakA*-up-R	GGCTGATCTGACCAGTTGCC
C-*SakA*-down-F	GGCTGTGTAGAAGTACTCGCC

**Table 2:** Quantitative Real-Time PCR primer sequences.

Primers	Sequence (5′ to 3′)
*AwGADPH*-RT-F	CGGCAAGAAGGTTCAGTT
*AwGADPH*-RT R	CTCGTTGGTGGTGAAGAC
*AwotaA*-RT-F	CGCCACGTCAATAAGTCTCGG
*AwotaA*-RT-R	GTATGGAGCGTGCAGATCTG
*AwotaY*-RT-F	ACACACAATCGTGAACAAGCG
*AwotaY*-RT-R	CATCCAGAAGTCCTCCACCG
*AwotaB*-RT-F	CTCGGCTACCTGCCTTCATG
*AwotaB*-RT-R	CAATGCCAACGCAATCAACG
*AwotaC*-RT-F	CGTGCGTTCAACTACCTAGACG
*AwotaC*-RT-R	CCTAGCCTCGGTGACATCAG
*AwotaR*1-RT-F	CCAGGACTCGTTCAGTCTCC
*AwotaR*1-RT-R	GAGCACCCTGCGACATCATG
*AwotaD*-RT-F	GCTGAACAACCAGAAGGAAGC
*AwotaD*-RT-R	GCCATCTCCTGGTTCATGACC

## Data Availability

Not applicable. Data is provided in the manuscript.

## References

[1] Malir F., Ostry V., Pfohl-Leszkowicz A., Malir J., Toman J. (2016). Ochratoxin A: 50 Years of Research. Toxins.

[2] Malir F., Ostry V., Novotna E. (2013). Toxicity of the mycotoxin ochratoxin A in the light of recent data. Toxin Rev..

[3] Pfohl-Leszkowicz A. (2009). Ochratoxin A and aristolochic acid involvement in nephropathies and associated urothelial tract tumours. Arh. Hig. Rada Toksikol..

[4] IARC (International Agency for Research on Cancer) (1993). Some Naturally Occurring Substances: Food Items and Constituents, Heterocyclic Aromatic Amines and Mycotoxins. IARC Monographs on the Evaluation of Carcinogenic Risks to Humans.

[5] Wang Y., Wang L., Wu F., Liu F., Wang Q., Zhang X., Selvaraj J.N., Zhao Y., Xing F., Yin W.-B. (2018). A Consensus Ochratoxin A Biosynthetic Pathway: Insights from the Genome Sequence of *Aspergillus ochraceus* and a Comparative Genomic Analysis. Appl. Environ. Microbiol..

[6] Gil-Serna J., Vazquez C., Sardinas N., Gonzalez-Jaen M.T., Patino B. (2009). Discrimination of the main Ochratoxin A-producing species in *Aspergillus* section *Circumdati* by specific PCR assays. Int. J. Food Microbiol..

[7] Visagie C.M., Varga J., Houbraken J., Meijer M., Kocsube S., Yilmaz N., Fotedar R., Seifert K.A., Frisvad J.C., Samson R.A. (2014). Ochratoxin production and taxonomy of the yellow aspergilli (*Aspergillus* section *Circumdati*). Stud. Mycol..

[8] Wang Y., Wang L., Liu F., Wang Q., Selvaraj J.N., Xing F., Zhao Y., Liu Y. (2016). Ochratoxin A Producing Fungi, Biosynthetic Pathway and Regulatory Mechanisms. Toxins.

[9] Gil-Serna J., Patino B., Cortes L., Gonzalez-Jaen M.T., Vazquez C. (2015). *Aspergillus steynii* and *Aspergillus westerdijkiae* as potential risk of OTA contamination in food products in warm climates. Int. J. Food Microbiol..

[10] Vipotnik Z., Rodriguez A., Rodrigues P. (2017). *Aspergillus westerdijkiae* as a major ochratoxin A risk in dry-cured ham based-media. Int. J. Food Microbiol..

[11] Tumukunde E., Li D., Qin L., Li Y., Shen J., Wang S., Yuan J. (2019). Osmotic-Adaptation Response of *sakA/hogA* Gene to Aflatoxin Biosynthesis, Morphology Development and Pathogenicity in *Aspergillus flavus*. Toxins.

[12] Lewis T.S., Shapiro P.S., Ahn N.G. (1998). Signal Transduction through MAP Kinase Cascades. Adv. Cancer Res..

[13] Chen Z., Gibson T.B., Robinson F., Silvestro L., Pearson G., Xu B.E., Wright A., Vanderbilt C., Cobb M.H. (2001). MAP Kinases. Chem. Rev..

[14] Takayama T., Yamamoto K., Saito H., Tatebayashi K. (2019). Interaction between the transmembrane domains of Sho1 and Opy2 enhances the signaling efficiency of the Hog1 MAP kinase cascade in *Saccharomyces cerevisiae*. PLoS ONE.

[15] Fang Y.L., Xia L.M., Wang P., Zhu L.H., Ye J.R., Huang L. (2018). The MAPKKK CgMck1 Is Required for Cell Wall Integrity, Appressorium Development, and Pathogenicity in *Colletotrichum gloeosporioides*. Genes.

[16] Liao X., Long X., He Q., Song M., Li X., Liu W., Zhang Y., Lin C., Miao W. (2021). Screening of binding proteins that interact with two components of the HOG MAPK pathway by the yeast two-hybrid method in *Colletotrichum siamense*. Eur. J. Plant Pathol..

[17] Stoll D., Schmidt-Heydt M., Geisen R. (2013). Differences in the regulation of ochratoxin A by the HOG pathway in *Penicillium* and *Aspergillus* in response to high osmolar environments. Toxins.

[18] Kawasaki L., Sánchez O., Shiozaki K., Aguirre J. (2002). SakA MAP kinase is involved in stress signal transduction, sexual development and spore viability in *Aspergillus nidulans*. Mol. Microbiol..

[19] Jaimes-Arroyo R., Lara-Rojas F., Bayram O., Valerius O., Braus G.H., Aguirre J. (2015). The SrkA Kinase Is Part of the SakA Mitogen-Activated Protein Kinase Interactome and Regulates Stress Responses and Development in *Aspergillus nidulans*. Eukaryot. Cell.

[20] Zhang Y., Choi Y.E., Zou X., Xu J.R. (2011). The *FvMK1* mitogen-activated protein kinase gene regulates conidiation, pathogenesis, and fumonisin production in *Fusarium verticillioides*. Fungal Genet. Biol..

[21] Nimmanee P., Woo P.C., Kummasook A., Vanittanakom N. (2015). Characterization of *sakA* gene from pathogenic dimorphic fungus *Penicillium marneffei*. Int. J. Med. Microbiol..

[22] Furukawa K., Hoshi Y., Maeda T., Nakajima T., Abe K. (2005). *Aspergillus nidulans* HOG pathway is activated only by two-component signalling pathway in response to osmotic stress. Mol. Microbiol..

[23] Graf E., Schmidt-Heydt M., Geisen R. (2012). HOG MAP kinase regulation of alternariol biosynthesis in *Alternaria alternata* is important for substrate colonization. Int. J. Food Microbiol..

[24] Igbalajobi O., Gao J., Fischer R. (2020). The HOG Pathway Plays Different Roles in Conidia and Hyphae During Virulence of *Alternaria alternata*. Mol. Plant-Microbe Interact..

[25] Manfiolli A.O., Mattos E.C., De Assis L.J., Silva L.P., Ulaş M., Brown N.A., Silva-Rocha R., Bayram Ö., Goldman G.H. (2019). *Aspergillus fumigatus* high osmolarity glycerol mitogen activated protein kinases SakA and MpkC physically interact during osmotic and cell wall stresses. Front. Microbiol..

[26] Garrido-Bazan V., Jaimes-Arroyo R., Sanchez O., Lara-Rojas F., Aguirre J. (2018). SakA and MpkC Stress MAPKs Show Opposite and Common Functions During Stress Responses and Development in *Aspergillus nidulans*. Front. Microbiol..

[27] Schmidt-Heydt M., Stoll D.A., Mrohs J., Geisen R. (2013). Intraspecific variability of HOG1 phosphorylation in *Penicillium verrucosum* reflects different adaptation levels to salt rich habitats. Int. J. Food Microbiol..

[28] Duran R., Cary J.W., Calvo A.M. (2010). Role of the osmotic stress regulatory pathway in morphogenesis and secondary metabolism in filamentous fungi. Toxins.

[29] Wang G., Li Y., Yang B., Li E., Wu W., Si P., Xing F. (2022). *AwAreA* Regulates Morphological Development, Ochratoxin A Production, and Fungal Pathogenicity of Food Spoilage Fungus *Aspergillus westerdijkiae* Revealed by an Efficient Gene Targeting System. Front. Microbiol..

[30] Nguyen A.N., Lee A., Place W., Shiozaki K. (2000). Multistep phosphorelay proteins transmit oxidative stress signals to the fission yeast stress-activated protein kinase. Mol. Biol. Cell.

[31] Buck V., Quinn J., Soto Pino T., Martin H., Saldanha J., Makino K., Morgan B.A., Millar J.B. (2001). Peroxide sensors for the fission yeast stress-activated mitogen-activated protein kinase pathway. Mol. Biol. Cell.

[32] Ma L., Li X., Xing F., Ma J., Ma X., Jiang Y. (2022). Fus3, as a Critical Kinase in MAPK Cascade, Regulates Aflatoxin Biosynthesis by Controlling the Substrate Supply in *Aspergillus flavus*, Rather than the Cluster Genes Modulation. Microbiol. Spectr..

[33] Rubenstein E.M., McCartney R.R., Zhang C., Shokat K.M., Shirra M.K., Arndt K.M., Schmidt M.C. (2008). Access denied: Snf1 activation loop phosphorylation is controlled by availability of the phosphorylated threonine 210 to the PP1 phosphatase. J. Biol. Chem..

[34] Román E., Arana D.M., Nombela C., Alonso-Monge R., Pla J. (2007). MAP kinase pathways as regulators of fungal virulence. Trends Microbiol..

[35] Mattos E.C., Silva L.P., Valero C., Castro P.A.d., Reis T.F.d., Ribeiro L.F.C., Marten M.R., Silva-Rocha R., Westmann C., Silva C.H.T.d.P.d. (2020). The *Aspergillus fumigatus* Phosphoproteome Reveals Roles of High-Osmolarity Glycerol Mitogen-Activated Protein Kinases in Promoting Cell Wall Damage and Caspofungin Tolerance. mBio.

[36] Bourret R.B., Kennedy E.N., Foster C.A., Sepúlveda V.E., Goldman W.E. (2021). A radical reimagining of fungal two-component regulatory systems. Trends Microbiol..

[37] Roman E., Alonso-Monge R., Gong Q., Li D., Calderone R., Pla J. (2009). The Cek1 MAPK is a short-lived protein regulated by quorum sensing in the fungal pathogen Candida albicans. FEMS Yeast Res..

[38] Frawley D., Bayram Ö. (2020). The pheromone response module, a mitogen-activated protein kinase pathway implicated in the regulation of fungal development, secondary metabolism and pathogenicity. Fungal Genet. Biol..

[39] Lara-Rojas F., Sanchez O., Kawasaki L., Aguirre J. (2011). *Aspergillus nidulans* transcription factor AtfA interacts with the MAPK SakA to regulate general stress responses, development and spore functions. Mol. Microbiol..

[40] Du C., Sarfati J., Latge J.P., Calderone R. (2006). The role of the *sakA (Hog1)* and *tcsB (sln1)* genes in the oxidant adaptation of *Aspergillus fumigatus*. Med. Mycol..

[41] Ochiai N., Tokai T., Nishiuchi T., Takahashi-Ando N., Fujimura M., Kimura M. (2007). Involvement of the osmosensor histidine kinase and osmotic stress-activated protein kinases in the regulation of secondary metabolism in *Fusarium graminearum*. Biochem. Biophys. Res. Commun..

[42] Alves de Castro P., Dos Reis T.F., Dolan S.K., Oliveira Manfiolli A., Brown N.A., Jones G.W., Doyle S., Riano-Pachon D.M., Squina F.M., Caldana C. (2016). The *Aspergillus fumigatus* SchA(SCH9) kinase modulates SakA(HOG1) MAP kinase activity and it is essential for virulence. Mol. Microbiol..

[43] Bruder Nascimento A.C.M.d.O., Dos Reis T.F., de Castro P.A., Hori J.I., Bom V.L.P., de Assis L.J., Ramalho L.N.Z., Rocha M.C., Malavazi I., Brown N.A. (2016). Mitogen activated protein kinases SakAHOG1 and MpkC collaborate for *Aspergillus fumigatus* virulence. Mol. Microbiol..

[44] Fabri J., Godoy N.L., Rocha M.C., Munshi M., Cocio T.A., von Zeska Kress M.R., Fill T.P., da Cunha A.F., Del Poeta M., Malavazi I. (2018). The AGC Kinase YpkA Regulates Sphingolipids Biosynthesis and Physically Interacts with SakA MAP Kinase in *Aspergillus fumigatus*. Front. Microbiol..

[45] Román E., Correia I., Prieto D., Alonso R., Pla J. (2020). The HOG MAPK pathway in Candida albicans: More than an osmosensing pathway. Int. Microbiol..

[46] Liang J., Slingerland J.M. (2003). Multiple roles of the PI3K/PKB (Akt) pathway in cell cycle progression. Cell Cycle.

[47] Hemmings B.A., Restuccia D.F. (2015). The PI3K-PKB/Akt Pathway. Cold Spring Harb. Perspect. Biol..

[48] Ikner A., Shiozaki K. (2005). Yeast signaling pathways in the oxidative stress response. Mutat. Res..

[49] Wang G., Wang Y., Yang B., Zhang C., Zhang H., Xing F., Liu Y. (2020). Carbon Catabolite Repression Gene *AoCreA* Regulates Morphological Development and Ochratoxin A Biosynthesis Responding to Carbon Sources in *Aspergillus ochraceus*. Toxins.

[50] Han X., Chakrabortti A., Zhu J., Liang Z.X., Li J. (2016). Sequencing and functional annotation of the whole genome of the filamentous fungus *Aspergillus westerdijkiae*. BMC Genom..

[51] Yu J.H., Hamari Z., Han K.H., Seo J.A., Reyes-Dominguez Y., Scazzocchio C. (2004). Double-joint PCR: A PCR-based molecular tool for gene manipulations in filamentous fungi. Fungal Genet. Biol..

[52] Wang G., Zhang H., Wang Y., Liu F., Li E., Ma J., Yang B., Zhang C., Li L., Liu Y. (2019). Requirement of LaeA, VeA, and VelB on Asexual Development, Ochratoxin A Biosynthesis, and Fungal Virulence in *Aspergillus ochraceus*. Front. Microbiol..

